# Isotope separation of ^176^Lu a precursor to ^177^Lu medical isotope using broadband lasers

**DOI:** 10.1038/s41598-021-85414-z

**Published:** 2021-03-17

**Authors:** M. V. Suryanarayana

**Affiliations:** Laser and Plasma Technology Division, Bhabha Atomic Research Centre, Visakhapatnam, Andhra Pradesh India

**Keywords:** Atomic and molecular physics, Quantum optics

## Abstract

A new photoionization scheme accessible by Rhodamine dye lasers is proposed for the isotope separation of ^176^Lu.

$$5d6s^{2}\,{^{2}D_{{3/2}}} (0.0\, {\text{cm}}^{{ - 1}} )\mathop{\longrightarrow}\limits^{{573.8130\, {\text{nm}}}}5d6s6p\,{^{4}F_{{3/2}}^{o}} \left( {17427.28\, {\text{cm}}^{{ - 1}} } \right)\mathop{\longrightarrow}\limits^{{560.3114\, {\text{nm}}}}$$

$$6s{6p}^{2}\,{^{4}{P}_{5/2}}\left(35274.5 \,{\text{cm}}^{-1}\right){\to } Autoionization\, State {\to }{Lu}^{+}$$

Optimum conditions for the laser isotope separation have been theoretically computed and compared with the previously reported work. The enrichment of ~ 63% can be obtained with > 22 mg/h production rate even when broadband lasers with bandwidth of 500 MHz are employed for the two step excitation. The simplified system requirements for the photoionization scheme combined with a high production rate of ^176^Lu than previously reported is expected to reduce the global shortage of ^176^Lu isotope for medical applications.

## Introduction

Chemistry which is a science of elements, compounds and their separation has immensely helped in the development of mankind. However, separation of isotopes remained a formidable challenge as it is technology intensive; therefore, it is largely limited only to the technologically advanced nations. Some of the isotopes of the low Z-elements could be separated relatively easily by the chemical methods by exploiting the difference in the kinetic isotope effect^1^. Since the kinetic isotope effect for mid-Z and high-Z elements gradually diminishes with increase in Z, it is necessary to switchover to physical methods for the efficient separation of isotopes of these elements. Isotope separation technology which is largely limited to the developed nations has been applied to the separation of actinides (particularly Uranium) due to its applications in the defence and nuclear industry^2^. However the real potential of isotope separation for the separation of rest of the isotopes remains unexploited due to limited access to this technology to most of the nations and the complexity of the systems involved.

Among the several methods of the separation of isotopes, gas centrifugation method has evolved as the most commercially viable method, however, apart from the technological challenges, the primary limitation, remains being highly capital intensive. On the other hand, Atomic Vapor Laser Isotope Separation (AVLIS) has evolved as an alternative technique to the gas centrifugation method. AVLIS has significant advantages over gas centrifugation such as being compact, less capital intensive and yielding in high separation factor. If AVLIS technique is appropriately deployed, these systems open up excellent opportunities for the separation of several isotopes other than Uranium.

In daily life, several radioisotopes are being used for medical applications and in particular for cancer diagnosis and therapy^3^. While many of them are produced as fission products in nuclear reactors; some can be obtained by the irradiation of the precursor (or parent) isotopes. The medical fraternity desires to use nuclear medicine in the highest possible radio-isotopic purity. This demand is normally not met for all the isotopes of nuclear medicine due to challenges in producing the precursor isotopes with a high level of isotopic purity (degree of enrichment) followed by full conversion into the medical isotopes upon irradiation. Among the several isotopes for nuclear medicine, ^177^Lu isotope has gained lots of attention due to its application in targeted radionuclide therapy^4^ (TRT). ^177^Lu is produced in the nuclear reactor by irradiation of its stable parent isotope ^176^Lu through the nuclear reaction $^{176}Lu\,{\mathop{\longrightarrow}\limits^{\sigma = 2090 b}} \,{}^{177}Lu$. ^177^Lu is a radioactive isotope having a half-life of 6.65 days, decays to ^177^Hf emitting β-particles with energies of 497 keV (76%), 384 keV (9.7%), 176 (12%). ^177^Hf which is formed in the nuclear excited states decays to the ground state, emitting low energy γ-radiation with energies of 208 keV and 113 keV. With mean β-particle penetration depths of 670 μm, ^177^Lu is very effective for the treatment of small tumors and metastatic lesions of small size. The low energy γ-radiation emitted by ^177^Hf is useful for imaging and the studies of bio-distribution and excretion kinetics. Further, large half-life of ^177^Lu enables shipment to long distances. For these reasons, ^177^Lu has evolved as the most preferred choice for the targeted radionuclide therapy. However, currently, there is a global shortage of ^177^Lu isotope primarily due to the shortage of its precursor which is enriched ^176^Lu isotope. Therefore, there is a need to enhance the production of this isotope to meet the global demand.

Separation of ^176^Lu (natural abundance = 2.59%) from the other natural major isotope ^175^Lu (natural abundance = 97.41%) through gas centrifugation is not possible as it has no volatile compounds^5^. Seemingly, AVLIS remains as the only available option for the enrichment of ^176^Lu. Kurchatov Institute^5–10^, Russia has extensively worked on the isotope separation of Lu isotopes. The experimental facilities required are technologically most demanding, particularly, with respect to the bandwidth of the excitation lasers, which has to be limited to < 100 MHz level. Such systems cannot be developed by the nations which are not technologically advanced, thus hampering large scale production of enriched ^176^Lu for medical applications. Therefore, search for suitable photoionization schemes with less stringent requirements and ability to produce at higher production rates is currently on.

In the present work, a new photoionization pathway is proposed for the isotope separation of ^176^Lu from natural Lu. The proposed photoionization scheme has been theoretically evaluated by computing ionization efficiency and isotope selectivity under various experimental conditions using density matrix formalism.

## Results and discussion

**Figure 1:**
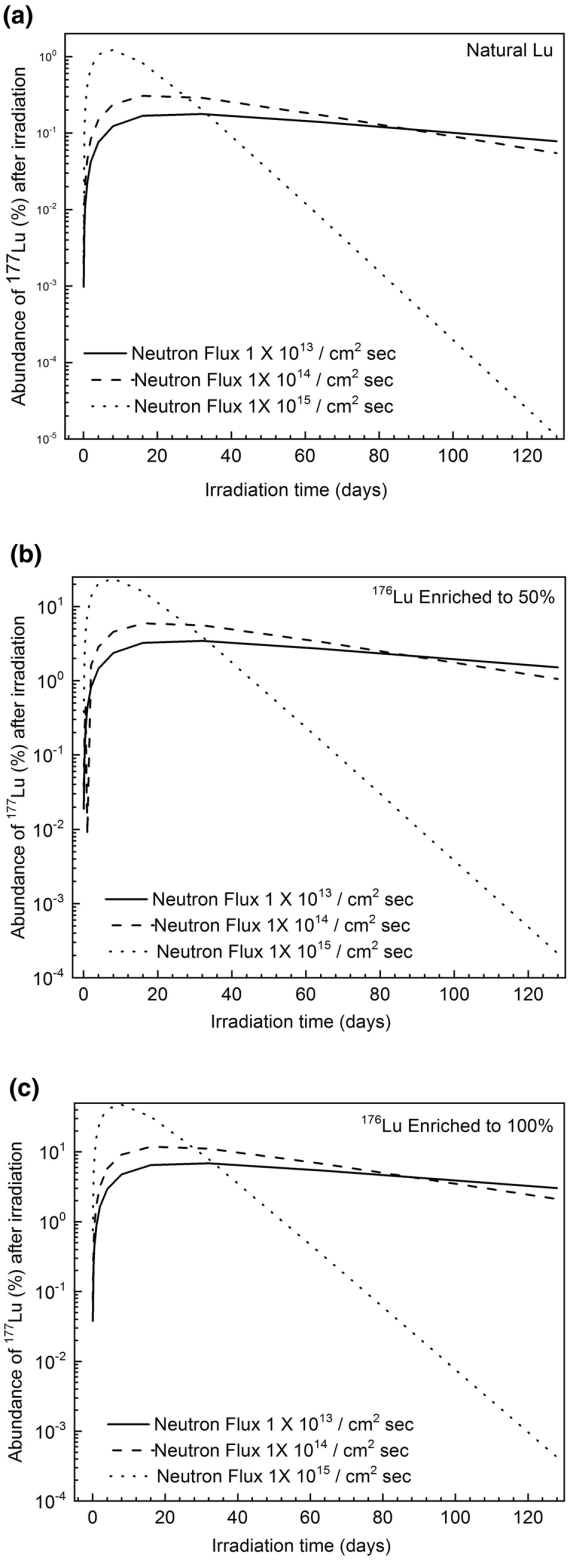
Percentage abundance of ^177^Lu after irradiation in a reactor with different nuclear fluxes for the cases of (**a**) natural Lu, (**b**) 50% enriched ^176^Lu and (**c**) 100% enriched ^176^Lu.

The primary limitation in direct irradiation of natural Lu for the production of ^177^Lu is the low natural abundance of its precursor ^176^Lu (2.59%). Upon direct irradiation of natural Lu in a medium to high flux reactors (neutron flux 10^14^–10^15^ neutrons/cm^2^-s), the content of ^177^Lu varies between 0.3 and 1.2% in the irradiated sample (Fig. 1a). When 50% enriched ^176^Lu is irradiated, it produces ^177^Lu whose content varies between 6 and 24% (Fig. 1b) in the irradiated sample. Further enrichment of ^176^Lu does not result in significant improvement in ^177^Lu content upon irradiation. For example, 100% enriched ^176^Lu produces ^177^Lu whose content varies between 12 and 47% (Fig. 1c). Therefore, enrichment of ^176^Lu to the degree of > 50% can be considered as optimum.

Density matrix formalism provides most accurate description of the laser-atom interaction^11^. Lineshapes arising in double-resonance^12^ and triple resonance ionization^13^ of atoms have been investigated in detail. Recently, investigation of the following photoionization pathway has been carried out for the ionization efficiency and degree of enrichment of ^177^Lu under various conditions.

### 540–535 nm scheme

$$5d6{s}\,^{2}\,{^{2}{D}_{3/2}} (0.0 \,{\text{cm}}^{-1}) {\mathop{\longrightarrow}\limits^{540.4068 \,\text{nm}}}\,5d6s6p {}\,{^{4}{F}_{5/2}^{o}}\left(18504.58\, {\text{cm}}^{-1}\right)\,{\mathop{\longrightarrow}\limits^{535.0626 \,\text{nm}}}$$

$$5d6s7s\,{^{4}{D}_{3/2}}\left(37193.98 \,\text{cm}^{-1}\right){\mathop{\longrightarrow}\limits^{618.0061 \,\text{nm}}} 53375\, \text{cm}^{-1}Autoionization\, State {\to }{Lu}^{+}$$

The results obtained were in good agreement with the experimental results^14^. The optimum conditions for the isotope selective photoionization of ^176^Lu have been derived. The primary limitation of the scheme for the production of enriched ^176^Lu is the utilization of narrowband lasers with bandwidth < 100 MHz. Moreover, incorporation of additional apertures along the atomic beam axis is required to limit the Doppler broadening of the atomic beam. This also causes reduction in the throughput of the enriched isotope.

From the energy levels of Lu, it is possible to formulate several photoionization pathways, among them; the following photoionization pathway seems to be particularly promising.

### 573–560 nm scheme

$$5{\mathrm{d}}6{\mathrm{s}}^{2}\,{^{2}{\mathrm{D}}_{3/2}} (0.0\, {\mathrm{cm}}^{-1}) {\mathop{\longrightarrow}\limits^{573.8130{\mathrm{ nm}}}} 5{\mathrm{d}}6{\mathrm{s}}6{\mathrm{p}}\,{^{4}{\mathrm{F}}_{3/2}^{\mathrm{o}}}\left(17427.28 \,{\mathrm{cm}}^{-1}\right) {\mathop{\longrightarrow}\limits^{560.3114\,{\text{nm}}}}$$

$$6{\mathrm{s}}{6{\mathrm{p}}}^{2}\,{^{4} {\mathrm{P}}_{5/2}} \left(35274.5 \,{\mathrm{cm}}^{-1}\right){\to }{\text{ Autoionization State }}{\to }{\mathrm{Lu}}^{+}$$

This photoionization pathway has a number of advantages over the photoionization scheme reported previously^5–10^. They are,

1. The wavelengths of the excitation lasers are easily accessible by high conversion efficiency Rhodamine dye lasers.
2. The large separation between hyperfine components of the Lu isotopes than previously reported enables better enrichment.
3. Possibility of employing relatively broadband lasers for the isotope separation minimises the stringent bandwidth requirements of the excitation lasers.

The hyperfine structure constants of Lu isotopes were measured by Brenner et al.^15^ and Kuhnert et al.^16^ for the 0.0 cm^−1^ and 17,427.28 cm^−1^ levels. Vergès and Wyart^17^ have measured hyperfine structure constants of ^175^Lu isotope for the 35,274.5 cm^−1^ level using Fourier Transform Infrared Spectrometry, whereas the HFS constants for the ^176^Lu have not been reported so far. In the absence of hyperfine anomalies^15,18^, the relationship between the hyperfine structure constants can be expressed as

1 $$\frac{{A}_{176}}{{A}_{175}}= \frac{{\mu }_{176} \times { I}_{175}}{{\mu }_{175} \times { I}_{176}}=\frac{3.1692 \times 3.5}{2.2325 \times 7}=0.71$$

2 $$\frac{{B}_{176}}{{B}_{175}}= \frac{{ Q}_{176}}{{ Q}_{175}}=\frac{4.92}{3.49}=1.41$$

where A, B are the magnetic dipole and electric quadrupole constants; µ is the magnetic moment, I is the nuclear spin and Q is the quadrupole moment. The hyperfine structure constants for the ^176^Lu were calculated to be A = 1372.9 MHz and B = − 3538.06 MHz for the 35,274.5 cm^−1^ level (Table 1).

**Table 1:** HFS coupling constants of lutetium energy levels.

Energy level	^175^Lu		^176^Lu		References
	A (MHz)	B (MHz)	A (MHz)	B (MHz)	
5d6s^2^ ^2^D_3/2_ (0.0 cm^−1^)	194.331617	1511.398650	137.920537	2132.296936	^15^
5d6s6p ^4^F^0^_3/2_ (17,427.28 cm^−1^)	− 924.82	1766.8	− 651.47	2494.2	^16^
6s6p^2 4^P_5/2_ (35,274.5 cm^−1^)	64.5^#^ 1933.66	− 83.70^#^ − 2509.26	1372.9 (calculated)	− 3538.06 (calculated)	^17^

^#^— Value in mK.

Based on the spectroscopic selection rules, a total of 30 hyperfine excitation pathways are possible in the step wise excitation scheme each denoted by a serial number and have been listed in Table 2 along with their resonance frequencies. Among them, the hyperfine excitation pathway 17/2–17/2–19/2 of ^176^Lu (listed in serial no 3 in Table 2) lies far away from the hyperfine pathways of the interfering ^175^Lu isotope. Isotope separation of ^176^Lu can be accomplished by ionization through the 17/2–17/2–19/2 hyperfine excitation pathway.

**Table 2:** A table of hyperfine excitation pathways of ^175^Lu and ^176^Lu of the photoionization (the frequency position is with reference to the centre of gravity of ^176^Lu isotope).

S no.	^175^Lu						^176^Lu					
	F1–F2–F3	Frequency Position of the first hyperfine transition (MHz)	Normalised Intensity	Frequency position of the second hyperfine transition (MHz)	Normalised intensity	Sum two photon frequency position (MHz)	F1–F2–F3	Frequency position of the first hyperfine transition (MHz)	Normalised intensity	Frequency Position of the second hyperfine transition (MHz)	Normalised intensity	Sum two photon frequency position (MHz)
1	5–5–6	− 5418.3	100.0	815.6	3.2	− 4996.1	17/2–17/2–15/2	− 8198.1	100.0	6483.0	5.4	− 1715.1
2	5–5–4	− 5418.3	100.0	10,394.3	23.7	4582.6	17/2–17/2–17/2	− 8198.1	100.0	17,756.1	29.6	9557.9
3	5–5–5	− 5418.3	100.0	20,705.8	100.0	14,894.1	17/2–17/2–19/2	− 8198.1	100.0	29,358.1	100.0	21,160.0
4	4–5–4	− 3367.1	38.9	815.6	3.2	− 2944.8	15/2–17/2–15/2	− 5731.2	49.1	6483.0	5.4	751.8
5	4–5–5	− 3367.1	38.9	10,394.3	23.7	6633.9	15/2–17/2–17/2	− 5731.2	49.1	17,756.1	29.6	12,024.9
6	4–5–6	− 3367.1	38.9	20,705.8	100.0	16,945.4	15/2–17/2–19/2	− 5731.2	49.1	29,358.1	100.0	23,626.9
7	5–4–3	− 2056.2	38.9	− 10,854.6	8.7	− 13,304.2	17/2–15/2–13/2	− 4175.0	49.1	− 8186.8	15.6	− 12,361.8
8	5–4–4	− 2056.2	38.9	− 2546.5	34.3	− 4996.1	17/2–15/2–15/2	− 4175.0	49.1	2459.8	44.0	− 1715.1
9	5–4–5	− 2056.2	38.9	7032.2	60.9	4582.6	17/2–15/2–17/2	− 4175.0	49.1	13,732.9	60.4	9557.9
10	4–4–3	− 5.0	24.2	− 10,854.6	8.7	− 11,253.0	15/2–15/2–13/2	− 1708.0	18.3	− 8186.8	15.6	− 9894.9
11	4–4–4	− 5.0	24.2	− 2546.5	34.3	− 2944.8	15/2–15/2–15/2	− 1708.0	18.3	2459.8	44.0	751.8
12	4–4–5	− 5.0	24.2	7032.2	60.9	6633.9	15/2–15/2–17/2	− 1708.0	18.3	13,732.9	60.4	12,024.9
13	3–4–3	340.5	50.5	− 10,854.6	8.7	− 10,907.5	13/2–15/2–13/2	− 937.2	65.1	− 8186.8	15.6	− 9124.1
14	3–4–4	340.5	50.5	− 2546.5	34.3	− 2599.3	13/2–15/2–15/2	− 937.2	65.1	2459.8	44.0	1522.6
15	3–4–5	340.5	50.5	7032.2	60.9	6979.4	13/2–15/2–17/2	− 937.2	65.1	13,732.9	60.4	12,795.7
16	2–3–2	4048.0	37.9	− 21,666.2	15.4	− 18,011.6	15/2–13/2–11/2	3486.3	65.1	− 23,139.0	30.5	− 19,652.7
17	2–3–3	4048.0	37.9	− 15,058.7	33.7	− 11,404.1	15/2–13/2–13/2	3486.3	65.1	− 13,381.2	43.9	− 9894.9
18	2–3–4	4048.0	37.9	− 6750.5	31.7	− 3095.9	15/2–13/2–15/2	3486.3	65.1	− 2734.5	30.6	751.8
19	4–3–2	4199.1	50.5	− 21,666.2	15.4	− 17,860.5	11/2–13/2–11/2	3859.0	48.9	− 23,139.0	30.5	− 19,280.0
20	4–3–3	4199.1	50.5	− 15,058.7	33.7	− 11,253.0	11/2–13/2–13/2	3859.0	48.9	− 13,381.2	43.9	− 9522.2
21	4–3–4	4199.1	50.5	− 6750.5	31.7	− 2944.8	11/2–13/2–15/2	3859.0	48.9	− 2734.5	30.6	1124.5
22	3–3–2	4544.6	0.0	− 21,666.2	15.4	− 17,515.0	13/2–13/2–11/2	4257.1	1.9	− 23,139.0	30.5	− 18,881.9
23	3–3–3	4544.6	0.0	− 15,058.7	33.7	− 10,907.5	13/2–13/2–13/2	4257.1	1.9	− 13,381.2	43.9	− 9124.1
24	3–3–4	4544.6	0.0	− 6750.5	31.7	− 2599.3	13/2–13/2–15/2	4257.1	1.9	− 2734.5	30.6	1522.6
25	2–2–1	8084.5	25.3	− 30,287.0	23.1	− 22,595.9	11/2–11/2–9/2	9607.9	50.5	− 37,529.5	50.0	− 27,921.5
26	2–2–2	8084.5	25.3	− 25,702.7	23.1	− 18,011.6	11/2–11/2–11/2	9607.9	50.5	− 28,887.9	29.5	− 19,280.0
27	2–2–3	8084.5	25.3	− 19,095.2	11.5	− 11,404.1	11/2–11/2–13/2	9607.9	50.5	− 19,130.1	10.5	− 9522.2
28	3–2–1	8581.1	37.9	− 30,287.0	23.1	− 22,099.3	13/2–11/2–9/2	10,006.0	48.9	− 37,529.5	50.0	− 27,523.4
29	3–2–2	8581.1	37.9	− 25,702.7	23.1	− 17,515.0	13/2–11/2–11/2	10,006.0	48.9	− 28,887.9	29.5	− 18,881.9
30	3–2–3	8581.1	37.9	− 19,095.2	11.5	− 10,907.5	13/2–11/2–13/2	10,006.0	48.9	− 19,130.1	10.5	− 9124.1

In an AVLIS process, when the first and second excitation lasers are tuned to the frequencies ν_1_ and ν_2_ respectively, isotope selectivity is defined as

3 $${\text{Isotope Selectivity S }}({\upnu }_{1},{\upnu }_{2})=\left(\frac{{\upeta }_{{176}_{\mathrm{Lu}}}}{{\upeta }_{{175}_{\mathrm{Lu}}}}\right)$$

where η is the ionization efficiency of the isotope.

For the case, wherein the abundance of the isotopes is not equal, one need to normalize the selectivity with the abundance of the constituent isotopes; thus, isotope ratio enhancement factor is defined as

4 $${\text{Isotope Ratio Enhancement Factor IRE }}({\upnu }_{1},{\upnu }_{2})=\left(\frac{{\upeta }_{{176}_{\mathrm{Lu}}}}{{\upeta }_{{175}_{\mathrm{Lu}}}}\right)*\left(\frac{{\mathrm{A}}_{{176}_{\mathrm{Lu}}}}{{\mathrm{A}}_{{175}_{\mathrm{Lu}}}}\right)$$

Degree of enrichment after laser interaction is calculated using the expression

5 $${\text{Degree of enrichment }}(\mathrm{\%}) =\left\{\frac{{\upeta }_{{176}_{\mathrm{Lu}}}{*\mathrm{A}}_{{176}_{\mathrm{Lu}}}}{\left({\upeta }_{{175}_{\mathrm{Lu}}}{*\mathrm{A}}_{{175}_{\mathrm{Lu}}}+{\upeta }_{{176}_{\mathrm{Lu}}}{*\mathrm{A}}_{{176}_{\mathrm{Lu}}}\right)}\right\}*100$$

**Figure 2:**
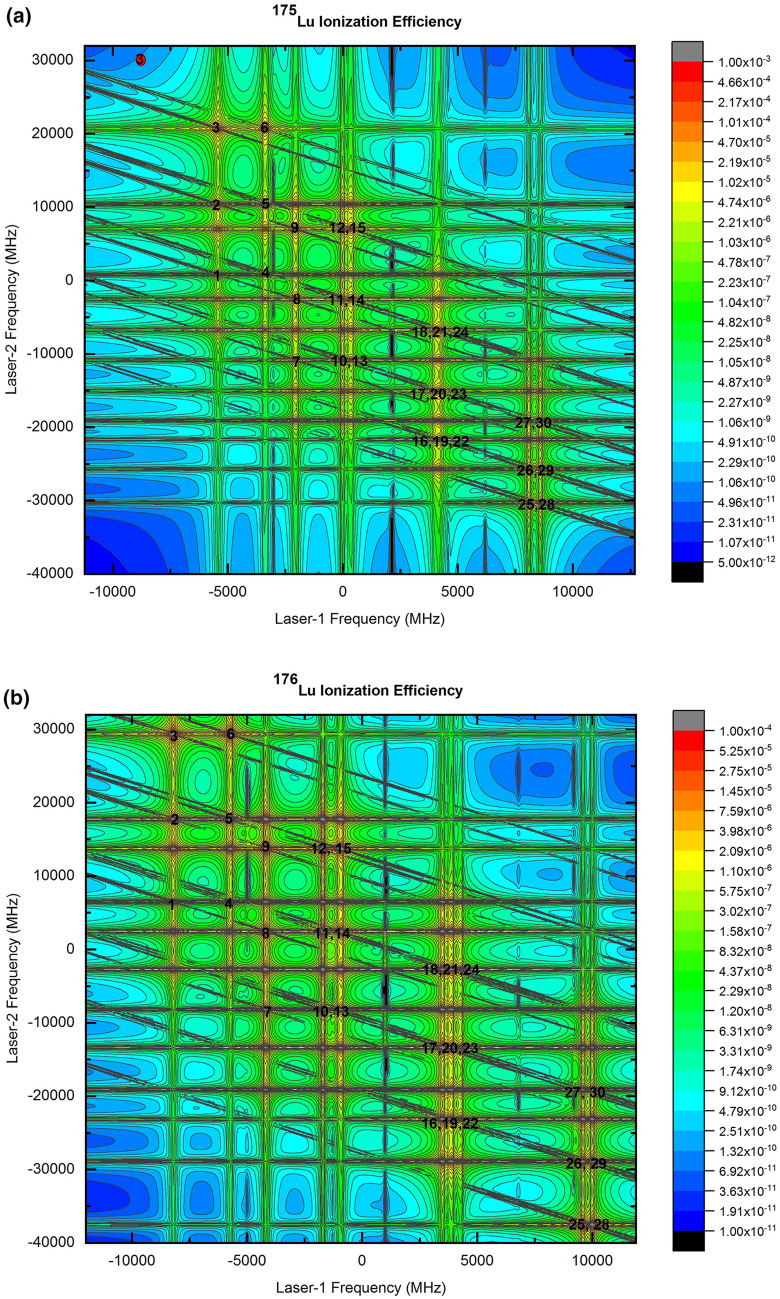
Two dimensional contour of the ionization efficiency of (**a**) ^175^Lu and (**b**) and ^176^Lu for the Doppler free condition and for a laser peak power density of 10 W/cm^2^ and a bandwidth of 10 MHz for the excitation lasers. The resonance frequency positions of the hyperfine excitation pathways are numbered as per the Table 2. The resonance frequency position of the 17/2–17/2–19/2 of the ^176^Lu is shown as “filled circle” in (**a**).

Two dimensional contour plots of the ionization efficiency of Lu isotopes are plotted in Fig. 2. The resonance frequency positions of all the possible hyperfine excitation pathways are numbered as per the Table 2. Horizontal ridges correspond to the first step excitation and vertical ridges correspond to the second step excitation. Strong horizontal and vertical ridges arise due to the strong transitions of the first and second excitation steps. Enhanced ionization observed at the intersection of horizontal and vertical ridges correspond either to the hyperfine pathway (numbered as per the Table 2) or crossovers. Origin of such crossovers has been discussed in detail in a recent article^14^. The resonance position of the 17/2–17/2–19/2 of ^176^Lu has been shown as a “filled circle” in the ^175^Lu two-dimensional contour plot in Fig. 2a.

The diagonal ridges correspond to the coherent two photon ionization pathway which is observed when the sum total energy of the two photons is equal to the energy of the upper level. In stepwise excitation schemes, coherent two photon ionization can be observed for the detuning (from the intermediate level) values matching the condition Δ_1_ = − Δ_2_. The probability of coherent two photon ionization decreases with the increase in the detuning (|Δ|) of the laser frequency from its resonance transition. For the strong transitions, as in the present case, the diagonal ridge can be observed even for large detuning (Fig. 2).

Diagonal ridges corresponding to the coherent two photon ionization of ^175^Lu hyperfine excitation pathways numbered as 3 and 6 closely pass through the 17/2–17/2–19/2 hyperfine pathway of ^176^Lu (Fig. 2a). The two photon sum frequency of 17/2–17/2–19/2 hyperfine pathway of ^176^Lu is − 8198.1 MHz + 29,358.1 MHz = 21,160.0 MHz (Table 2). The two photon sum frequencies of the hyperfine excitation pathways of ^175^Lu numbered 3 and 6 ranges between 14,894.1 MHz to 16,945.4 MHz thus have considerable impact on the degree of enrichment of the AVLIS process due to coherent two photon ionization.

Computation of ionization efficiencies and isotope selectivity have been calculated varying the peak power density of excitation and ionization lasers for the Doppler free case and optimum conditions for the enrichment are tabulated in Table 3. From the Table 3, the limit for the bandwidth of the excitation lasers is found to be 500 MHz for obtaining the degree of enrichment of ~ 66%.

**Table 3:** Variation in the selectivity, isotope ratio enhancement (IRE) factor and degree of enrichment of ^176^Lu (for the Doppler free case) with the increase in the laser bandwidth.

Laser bandwidth (MHz)	Peak power density (W/cm^2^)			η_176_	Selectivity	IRE (natural Lu)	Degree of enrichment %
	573.8130 nm	560.3114 nm	Ionization laser				
100	40	20	16,000	0.294	9784	261	99.6
250	80	20	18,000	0.197	653	17	94.4
500	100	40	22,000	0.179	73	2	65.6

Numerical computations of ionization efficiency and degree of enrichment have been carried out for various experimental parameters such as bandwidth of the excitation lasers, peak power density of excitation and ionization lasers and half angular divergence limit to the atomic beam with a limit to the ionization efficiency set to ~ 0.2 and results are tabulated in Table 4. The ionization efficiency of 0.2 corresponds to the ionization of 80% of population available at the ground hyperfine level 17/2. In order to compare the present results with the previously reported data^5,14^, production rates have been calculated for the atomic number density of 5 × 10^14^ of Lu corresponding to the number density of 1.3 × 10^13^ for ^176^Lu. Further, the laser-atom interaction length is considered as 200 mm with a diameter of 10 mm. It can immediately be observed that for the case of lasers with a bandwidth of 100 MHz, the degree of enrichment reaches to > 99% for all angular divergence values. While for the case of laser bandwidth of 250 MHz, the degree of enrichment degrades to ~ 94%; for higher angular divergence values of the atomic beam, the degree of enrichment marginally deteriorates to ~ 93%. The increase in the angular divergence of the atomic beam has little impact on the degree of enrichment, which implies that under such conditions, larger production rates can be obtained without significant deterioration in the degree of enrichment. Even in the case of lasers with bandwidth of 500 MHz, a degree of enrichment of ~ 63% can be achieved with no limits on the angular divergence.

**Table 4:** A table of degree of enrichment and production rates of ^176^Lu for various experimental parameters.

Laser bandwidth (MHz)	Half angle divergence	Co-propagating excitation laser beams					Counter-propagating excitation laser beams				
		η_176_	Selectivity	IRE (natural Lu)	Degree of enrichment %	Production Rate (mg/h)	η_176_	Selectivity	IRE (natural Lu)	Degree of enrichment %	Production Rate (mg/h)
100	Doppler free (0°)	0.294	9784	261	99.6	–	0.294	9784	261	99.6	–
	5°	0.263	8749	233	99.6	3.5	0.263	8743	233	99.6	3.5
	15°	0.238	7894	211	99.5	9.6	0.239	7893	211	99.5	9.7
	30°	0.191	6284	168	99.4	15.3	0.193	6284	168	99.4	15.5
	45°	0.160	5240	140	99.3	18.4	0.163	5212	139	99.3	18.8
	60°	0.145	4697	125	99.2	19.5	0.148	4641	124	99.2	19.9
	75°	0.138	4446	119	99.1	18.9	0.141	4371	117	99.1	19.3
	90°	0.137	4430	118	99.1	18.7	0.140	4367	117	99.1	19.1
250	Doppler free (0°)	0.197	653	17	94.4	–	0.197	653	17	94.4	–
	5°	0.197	651	17	94.4	2.7	0.197	650	17	94.4	2.7
	15°	0.191	631	17	94.3	7.7	0.191	630	17	94.2	7.7
	30°	0.178	584	16	93.8	14.3	0.178	578	15	93.8	14.3
	45°	0.165	538	14	93.3	19.0	0.165	528	14	93.2	19.0
	60°	0.156	506	14	92.9	21.0	0.156	493	13	92.8	21.0
	75°	0.151	490	13	92.7	20.6	0.152	475	13	92.5	20.8
	90°	0.151	489	13	92.7	20.6	0.151	475	13	92.5	20.6
500	Doppler free (0°)	0.179	73	2	65.6	–	0.179	73	2	65.6	–
	5°	0.179	73	2	65.6	2.4	0.179	73	2	65.6	2.4
	15°	0.177	73	2	65.4	7.2	0.177	72	2	65.3	7.2
	30°	0.173	71	2	64.8	13.9	0.173	70	2	64.6	13.9
	45°	0.169	69	2	64.1	19.5	0.169	68	2	63.8	19.5
	60°	0.165	67	2	63.6	22.2	0.165	66	2	63.1	22.2
	75°	0.163	66	2	63.3	22.3	0.163	65	2	62.6	22.3
	90°	0.163	66	2	63.3	22.3	0.163	65	2	62.7	22.3

Production rates are calculated for the atomic number density of 5 × 10^14^ of Lu. (corresponding to the number density 1.3 × 10^13^ of ^176^Lu); length of the laser-atom interaction region 200 mm, laser beam diameter of 10 mm. Peak power density of the first, second excitation lasers and ionization laser for the 100 MHz case are 40, 20, 16,000 W/cm^2^; for 250 MHz case are 80, 20, 18,000 W/cm^2^; for 500 MHz case are 100, 40, 22,000 W/cm^2^ respectively.

The present results are compared with the previously reported^5,14^ values in Table 5. For the case of lasers with bandwidth of 100 MHz, even for the maximum possible half angular divergence value (90°), the degree of enrichment is found to be > 99%, which is nearly 3 times higher than the previously reported 540–535 nm scheme by D’yachkov^5^ et al. It is not possible to enrich the ^176^Lu isotope > 5% using broadband lasers having a width of 500 MHz using the 540–535 nm photoionization scheme. However, lasers with bandwidth of 500 MHz can be used in the present 573–560 nm scheme and a degree of enrichment of ~ 63% can be achieved with a production rate > 22 mg / hour which is 6 times higher than the previously reported rate of 3.7 mg/h. Thus the proposed scheme enables production of ^176^Lu with higher production rate with relatively broad band lasers.

**Table 5:** Comparison of the photoionization schemes for the degree of enrichment and production rates of ^176^Lu for various experimental parameters.

Bandwidth (MHz)	Half angle divergence (degrees)	540–535 nm scheme				573–560 nm scheme^#^	
		D’yachkov et al.^5^		Previous work^14^		Present work	
		Production rate (mg/h)	Degree of enrichment (%)	Production rate (mg/h)	Degree of enrichment (%)	Production rate (mg/h)	Degree of enrichment (%)
100	90°	22.55	30.7	22.55	33.8–34.7	19.1	99.1
	5°	3.7	68.4	3.5	66.6–66.8	3.5	99.6
250	90°	–	–	–	–	20.6	92.5–92.7
500	5°	–	–	–	–	2.4	65.6
	45°	–	–	–	–	19.5	63.8–64.1
	90°	–	–	–	–	22.3	62.7–63.3

^#^See Table 4 for laser peak power density of the lasers.

### Effect of unknown parameters

In atomic vapor laser isotope separation process, all the atomic parameters such as isotope shifts, hyperfine structures, lifetimes, branching ratios, decay to trapped, autoionization states and their cross-sections; laser parameters such as peak power density, pulse width, bandwidth, repetition frequency; source parameters such as temperature, angular divergence all influence the overall degree of enrichment and the production rate. For the case of the 573–560 nm scheme, some of the spectroscopic parameters have not been measured so far and hence are not known. The effect of these parameters on the degree of enrichment and the resultant production rates is discussed below.

- Isotope shift for the 560 nm transition

Isotope shift for the $5d6s6p\,{^{4}{F}_{3/2}^{o}}\left(17427.28 \,\text{cm}^{-1}\right)\,{\mathop{\longrightarrow}\limits^{{560.3114 \,\text{nm}}}}\, 6s{6p}^{2}\,{^{4}{P}_{5/2}}\left(35274.5 \,\text{cm}^{-1}\right)$ transition has not been reported so far. Isotope shift of a transition comprises of field shift and the mass shift components which can be expressed as^19^

$$\delta \gamma \approx F. {\delta \langle {r}^{2}\rangle }^{\mathrm{175,176}}+M.\left(\frac{{M}^{175}- {M}^{176}}{{M}^{175}.{M}^{176}}\right)$$

where F, M are the field shift and mass shift parameters, ${\delta \langle {r}^{2}\rangle }^{\mathrm{175,176}}$ is the difference in the mean square nuclear charge radii.

For the case of Lutetium isotopes, the difference in the mean square nuclear charge radii ${\delta \langle {r}^{2}\rangle }^{\mathrm{175,176}}$ = 0.041 fm^2^ is small^19^, resulting in small field shift. The mass shift is also small owing to the small value of the factor $\left(\frac{{M}^{175}- {M}^{176}}{{M}^{175}.{M}^{176}}\right)$. As a result, the isotope shift between ^176^ and ^177^Lu is small *i.e.,* about ~ 100 MHz. Large isotopic selectivity of the present scheme is due to the large spread of the hyperfine spectrum of ^176^Lu, therefore, the influence of the variation in isotope shift is not of much significance.

(b) Einstein’s coefficient for the $5{{d}}6{{s}}6{{p}}\,{^{4}{{{F}}}_{3/2}^{{{o}}}}\left(17427.28\,{{{c}}{{m}}}^{-1}\right)\,{\mathop{\longrightarrow}\limits^{{560.3114 {{n}}{{m}}}}}\,{\to } 6{{s}}{6{{p}}}\,^{2}\,{^{4}{{{P}}}_{5/2}}\,\left(35274.5\,{{\text{cm}}}^{-1}\right)$ transition

Einstein’s A coefficient for the transition has not reported so far. However, the typical values reported for the ^4^P fine structure level were reported to be in the range of 3 × 10^6^ to 2 × 10^7^. A mean value of 1.3 × 10^7^ has been taken for the computations. Due to this uncertainty in the value of Einstein’s A coefficient, the optimum peak power density of the second excitation transition may vary between 20 and 97 W/cm^2^ which can easily be achieved for the pulsed lasers. Due to the large spread of the hyperfine spectrum of ^176^Lu isotope and the separation of its 17/2–17/2–19/2 hyperfine excitation pathway from the ^175^Lu spectrum, the power broadening (due to increase in the laser peak power density) varies the ionization efficiency rather than the selectivity.

(c) Autoionization states and cross-sections

The non-availability of data on the autoionization states and their ionization cross-sections is the primary impediment to the proposed 573–560 nm scheme. Most of the previous work^20,21^ carried out have reported the data pertaining to the Rydberg series and autoionization states originating from the 5d6s6p ^2^D^o^_3/2,5/2_, 5d6s6p ^4^D^o^_3/2,5/2_ and 6s^2^nd ^2^D_3/2,5/2_, 6s^2^ns ^2^S_1/2_ states. D’yachkov et al.^5,10^ have found a strong autoionization level connecting to the 5d6s7s ^4^D_3/2_ upper level of the 540–535 nm scheme. Further experimental studies on the search for suitable autoionization states connecting to the 6s6p^2 4^P_5/2_ upper level of the 573–560 nm scheme is necessary.

## Conclusion

A new photoionization pathway has been proposed for the isotope separation of ^176^Lu precursor isotope. The optimum conditions for the isotope separation of ^176^Lu have been obtained through density matrix formalism. It has been shown that it is possible to obtain an enrichment of > 99% with high production rates of 19 mg/h when the excitation lasers with bandwidth of 100 MHz are employed. Even when broadband dye lasers of bandwidth of 500 MHz are used, the degree of enrichment of ~ 63% can be obtained with a production rate of > 22 mg/h. This significantly eases the restrictions on the bandwidth of the excitation lasers and Doppler broadening enabling high production rates than previously reported, thus expected to minimise the global shortage of the^176^Lu precursor isotope for the production of the ^177^Lu medical isotope.
